# Systematic review of interventions to reduce illicit drug use in female drug-dependent street sex workers

**DOI:** 10.1136/bmjopen-2015-009238

**Published:** 2015-11-18

**Authors:** Nikki Jeal, John Macleod, Katrina Turner, Chris Salisbury

**Affiliations:** Centre for Academic Primary Care School of Social & Community medicine, University of Bristol, Bristol, UK

## Abstract

**Objectives:**

Most female street-based sex workers (SSWs) are drug users and this group experience particularly poor outcomes in achieving and maintaining abstinence. In 2010 the UK adopted a recovery-orientated Drug Strategy. This strategy did not specifically highlight the complex drug treatment needs of SSWs. Therefore we sought to synthesise and critically appraise existing evidence of interventions to reduce illicit drug use in this group, in order to guide service change toward better provision for the drug treatment needs of SSWs.

**Methods:**

A systematic review of evidence on the effectiveness of interventions to reduce illicit drug use in female SSWs. Following the PRISMA guidelines, a structured search strategy was used. Searches included databases, organisational and government websites to identify published and grey literature, as well as contacting experts in the field, and hand-searching reference lists and journals.

**Results:**

Six studies, one experimental and five observational, were identified which met review inclusion criteria. Intervention approaches evaluated included substitute prescribing, educational sessions and motivational interviewing. All studies reported a positive intervention effect but the five observational studies were all subject to a relatively high risk of bias. By contrast, the experimental study provided little or no evidence of positive effect (OR for reduction of illicit drug in intervention compared to controls 1.17 95%CI 0.84–1.66 at 3 months and 1.14 (95% CI 0.8 to 1.61) at 6 months follow-up). All six studies described challenges and solutions to study recruitment, retention and follow-up, which were influenced by issues affecting SSWs’ health and social stability.

**Conclusions:**

There is currently no strong evidence for effectiveness of interventions to reduce illicit drug use in female SSWs with problematic drug use. Thus, the development and robust evaluation of effective interventions should be a priority if recovery-orientated goals are to become more achievable for this group.

**Strengths and limitations of this study**

- This is the first systematic review looking at interventions specifically targeting levels of illicit drug use, as opposed to wider harm reduction, in street-based sex workers (SSWs).
- A very inclusive approach to interventions was used to reflect the complex influences on SSWs’ health and service use.
- This review is addressing an important question in response to UK government health policy.
- Data relating to the challenges of research with a hard to reach population are included in the data synthesis.
- The small number of studies, with different study designs, interventions and contexts, makes it difficult to draw clear conclusions except that evidence of success from any particular strategy is lacking.

## Introduction

There is a need to focus on reducing illicit drug use in street-based sex workers (SSWs) as drug policy aims shift from a pragmatic harm reduction approach, tolerant of continuing drug use, as long as this drug use is safer, to recovery-orientated services.^1^ Debate on the appropriateness of this reorientation continues,^2–4^ and there is a concern that current policy embodied in the 2010 UK Drug Strategy^5^ does not identify SSWs as a group with specific needs. This suggests an assumption that mainstream services will be expected to continue to meet the treatment needs of this vulnerable group.^6^

Compared to other drug users, SSWs have particularly complex health and social needs.^7^ The concurrent use of heroin and cocaine, the commonest drugs used by SSWs,^8^ is associated with the poorest treatment outcomes among drug service users.^9^ Their drug use is more prolific, they have higher levels of mortality and are less likely to achieve abstinence.^7^ They experience worse mental and physical health and are more likely to have a personal history of prior sexual and physical abuse.^10^ Behavioural effects of recent drug use increase vulnerability to violence and sexual risk-taking while working.^11^ These issues are further compounded by the fact that female drug users do not access drug services to the same extent as their male counterparts,^12^ and for female SSWs this is a particular problem.^13^ ^14^ Unstable social circumstances, including housing, are an additional influence on health and service use in this group.^15^

Progress has been made in terms of protecting the health of sex workers through the harm-reduction approach including the development of interventions effective in reducing transmission of HIV and other blood-borne viruses, reducing transmission of sexually transmitted infections (STIs) and increasing safer drug use practices.^16^ ^17^ This approach has often included an emphasis on the importance of involving sex workers in developing and delivering interventions and on improving sex workers’ rights.^18^ ^19^

Evidence of effectiveness aside, recent drug policy including that in the UK has moved away from a broader harm reduction approach to a narrower focus on ‘recovery’ based on the assumption that the most rational approach to the prevention of drug-related harm is the prevention of drug use. However, ostensibly rational, such an approach may not be effective in meeting the often complex needs of SSWs who use illicit drugs. To inform the drug policy debate we undertook a systematic review of evidence on the effectiveness of interventions to reduce problem drug use in SSWs.^20^

### Objective of the review

The primary objective of this review is to collate, summarise and critically appraise evidence of effects of interventions targeting illicit drug use in female SSWs. Levels of sex work and homelessness, where reported, are secondary outcomes as they are inextricably linked with problematic drug use and have a direct influence on SSWs’ health and use of health services.^15^

The review protocol has been submitted as an online supplementary file and is available from the corresponding author.

## Methods

### Search strategy

The search strategy was designed to identify published and unpublished studies in manuscripts, reports and literature available through relevant databases and organisation websites. Databases searched are outlined in box 1.The strategy was also designed to identify grey literature.

Box 1 **Sources of evidence**

Electronic databases searched

- AMED
- BIOSIS
- CINAHL
- Embase
- ERIC
- IBSS
- Medline
- PsychINFO
- Social Services Abstracts
- Sociological Abstracts
- Web of Science
- Pubmed*

*Theses databases*

- DARTEurope
- EThOS
- Index to Theses

Websites

Systematic reviews

- Cochrane Library
- Centre for Reviews and Dissemination
- Campbell library of systematic reviews

Other synthesis

- National Institute for Clinical Excellence
- National Treatment Agency
- Database of Public Health Intervention Reviews
- Scottish Intercollegiate Guideline Network

Organisations

- Home Office
- UK Network of Sex Work Projects
- Department of Health
- Cabinet Office

*Hand searching journals*

1. Drug and Alcohol Dependence
2. Journal of Substance Abuse Treatment
3. International Journal of Drug Policy

Hand searching bibliography of papers included in the review

Papers that cite papers included in the review

Experts contacted who responded

1. Wendee Weschberg (USA)
2. Christine Grella (USA)
3. Steffanie Strathdee (USA)
4. Susan Sherman (USA)
5. Matt Hickman (UK)
6. Hilary Surratt (USA)

Main searches were conducted to May 2013 and updated through Medline and Pubmed searches in January 2015. There were no language or publication status constraints on consideration for inclusion in the review. Manuscripts in languages other than English were translated and assessed. Six experts in the field from the USA and the UK responded to direct contact requesting suggestions of relevant studies.

Searches were not limited to index terms but included free text to increase their comprehensiveness. Search terms focusing on sex work and drug use were used. Owing to the unreliability of the classification and indexing of observational studies, no search restrictions relating to study design were imposed.^21^ The Medline search strategy (box 2) was adapted for other databases.

Box 2 **Electronic search strategy for Medline**

Medline on Ovid

1. prostitution
2. prostitut*.tw
3. sex adj1 work*.tw
4. substance-related disorders
5. amphetamine-related disorders
6. cocaine-related disorders
7. crack cocaine
8. heroin dependence
9. morphine dependence
10. opioid-related disorders
11. street drugs
12. substance abuse, intravenous
13. 1 or 2 or 3
14. 4 or 5 or 6 or 7 or 8 or 9 or 10 or 11 or 12
15. 13 and 14

### Inclusion and exclusion criteria

#### Studies

All quantitative study designs that deployed an intervention described as affecting levels of illicit drug use, as a primary or secondary outcome, were eligible for inclusion. Eligible study designs were randomised controlled trials (cross-over, cluster and stepped wedge), quasi experimental studies (non-randomised controlled studies, before and after studies and interrupted time series) and observational studies (cohort and case control studies). Case series and case reports were excluded due to their potential for bias.

#### Participants

Study inclusion criteria required at least 90% of the study population to be female. Participants were required to be currently using opiates and/or crack cocaine and currently involved in street-sex work as their principle sector of work. Current drug use was defined as use on more than one occasion in the last 30 days or 1 month. Current sex work was defined as having sold sex on the street with in the last 30 days or 1 month. Studies in which participants were currently incarcerated were excluded.

#### Interventions

Any intervention, with or without a comparator that considered levels of problem opiate or crack cocaine use, as an outcome, was included irrespective of route of drug use.

#### Outcomes

All studies were required to report a primary or secondary outcome measure of illicit drug use (heroin and/or crack cocaine). Data relating to the secondary outcomes of involvement in sex work and homelessness, were collected where available. No restriction was applied to timing of outcome assessment.

### Data extraction and quality assessment

Initial screening of titles and abstracts were undertaken by a single reviewer (NJ) against inclusion and exclusion criteria preagreed with the other review authors. Owing to inclusion of observational studies in the review, all studies that could not be confidently excluded on the abstract were included for screening of the full text manuscript. Screening of full text manuscripts for inclusion was undertaken independently by two screeners (NJ and David Burton (DB)). No disagreements on eligibility occurred.

Data were extracted onto a data extraction form based on the format in the Cochrane Handbook for Systematic Reviews of Interventions.^21^ Risk of bias was assessed using the approach outlined in the Cochrane Handbook for Systematic Reviews of Interventions^21^ and how the randomisation sequence was generated, how allocation was concealed, the integrity of blinding at outcome assessment, the completeness of outcome data, selective reporting and other potential sources of bias were considered. Findings were discussed with the other review authors before inclusion in the synthesis, and all eligible studies were included irrespective of their assessed risk of bias. The assessed risk of bias and characteristics of included studies informed the approach to data synthesis.

### Data synthesis

Initial tabulation was undertaken to assess comparability of outcome measures. Owing to the observational methodology, small numbers of study participants in all but one of the included studies and insufficiently similar study outcomes,^21^ it was not considered appropriate to undertake meta-analysis or to undertake statistical tests for heterogeneity.^21^

Data were managed by undertaking narrative synthesis.^22^ Studies were grouped and tabulated according to all variables considered likely to influence study outcomes and intervention effect. These were age of studies, intervention type, intervention focus and country where study was undertaken. Evidence for an intervention effect was considered across studies, in relation to primary and secondary outcomes,^23^ and with regard to direction, magnitude, strength and consistency.

Themes across studies that related specifically to development and implementation of the intervention, and undertaking and interpreting research with this population, were analysed using the software package NVivo.

## Results

**Figure 1:**
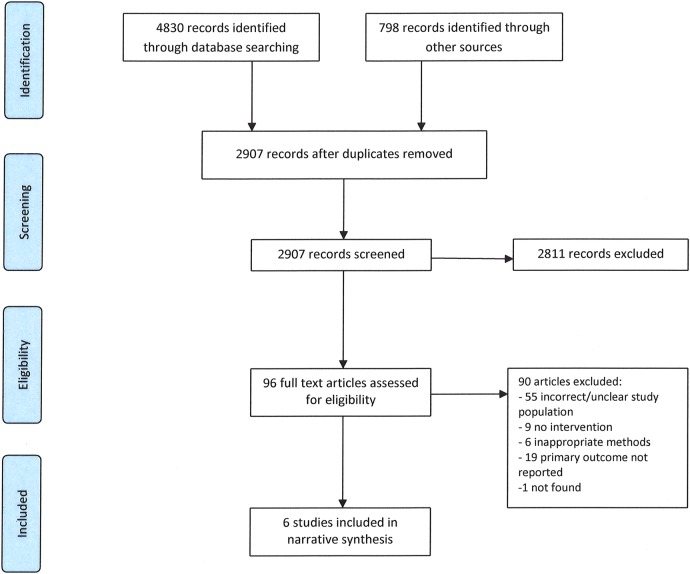
Prisma flowchart for systematic review of interventions to reduce illicit drug use in drug-dependent street sex workers.

A total of 2907 records were identified for screening of title and/or abstract. Of those, 96 records either appeared to meet the inclusion criteria or had insufficient information to make a decision. These 96 records were included for full text screening (figure 1).

Six studies were included in the review after full text screening.^24–29^ The characteristics of these studies are summarised in table 1.

**Table 1:** Included studies—characteristics and risks of bias

First author (year)	Country	Setting	Participants	Participant age	Study design	Follow-up	Risks of bias
Surrat (2010)	USA	Community	806 410 intervention 396 control	36.8 years (mean) (SD 8.2)	RCT	3 and 6 months	No detail of randomisation or allocation process No blinding of participants or personnel Self-reported non-blinded outcome measures Unclear whether analysis decisions were prospective eg, dichotomisation of outcome results Follow-up rates calculated by combining attendance at either of follow-up appointments which increased follow-up rates Intention to treat analysis not undertaken
Litchfield (2010)	UK	Community	34	Not stated	Before/after clinical records	12 months	All participants received intervention and no control group Self-reported non-blinded outcome measures Missing data not accounted for
Sherman (2006)	USA	Community	54	39 years (median) (IQR 34–45)	Before/after survey	3 months	All participants received intervention and no control group Self-reported and non-blinded outcome measures Missing data not accounted for
Yahne (2002)	USA	Community	27	37.8 years (mean) (SD 8.1)	Before/after survey	4 months	All participants received intervention and no control group Self-reported and non-blinded outcome measures Follow-up in settings likely to influence outcomes and act as confounder
Bellis (1993)	USA	Outpatient	41	31.8 years (mean)	Before/after	12 months	All participants received intervention and no control group Self-reported non-blinded outcome measures No data on participants lost to follow-up
Gunne (1986)	Sweden	In-patient	34	28.1 years (mean) (SD 5.8)	Before/after clinical records	1–220 months	All participants received intervention and no control group Self-reported non-blinded outcome measures

### Study design

One randomised controlled trial (RCT)^24^ and five before and after studies^25–29^ were included. There were 806 participants in the RCT and the number of participants in the observational studies ranged between 27 and 54.

### Participants

All participants were reported as current female SSWs and were using heroin or crack cocaine. Mean age of participants was given in four studies and median age in one. Average age of participants was lower in the older studies. A mean of 28.1 years (SD 5.8) was reported in the 1986 study^29^ compared to mid to late 30 s in the most recent studies. Intensity of participant involvement in sex work at study entry was difficult to compare across studies due to differing outcome measures and descriptors.

### Location

Four of the six studies were based in the USA,^24^ ^26–28^ one in the UK^25^ and one in Sweden.^29^

### Setting

The three substitute prescribing based studies^25^ ^28^ ^29^ were in clinical settings. The oldest 1986 study^29^ was in-patient based, the slightly later 1993 study^28^ outpatient based and the 2010 study^25^ based in a community clinic. All the non-clinical interventions were based in a community outreach setting.

### Interventions

Interventions were of three types: substitute prescribing based,^25^ ^28^ ^29^ educational^24^ ^26^ and motivational interviewing (MI).^27^

#### Substitute prescribing based

The three interventions which involved substitute prescribing^25^ ^28^ ^29^ were based on novel approaches to delivery of drug treatment and/or drug treatment services. The earliest study^29^ was published in 1986 and was based in Sweden where methadone was emerging as an alternative to in-patient detoxification, so the intervention was methadone-based substitute prescribing. The 1993 study^28^ was US-based^28^ where methadone was already an established method of treatment of opiate dependency and the intervention was provision of free methadone in a private health system. The 2010 study was UK-based (healthcare free at point of delivery with methadone routinely available) and looked at the effect of a sex worker-only clinic which ran within a general drug treatment service. All the interventions included provision of a broad range of healthcare alongside substitute medication.

#### Educational based

The educational interventions^24^ ^26^ adopted novel approaches to content and delivery of HIV risk reduction education. The Sherman 2006^26^ educational intervention included jewellery-making, marketing and selling, in addition to HIV risk reduction information in six highly structured 2 h sessions delivered over a 3-week period by a trained facilitator following a manual. The intervention aimed to increase knowledge of HIV/STIs and risk reduction, enhance self-efficacy in practicing safer sex, and reduce sexual and drug use risk-taking. It also aimed to increase negotiation and communication skills, and provide opportunity to practice those skills in role play. The jewellery-making training was intended to develop skills to provide an alternative to sex work for generating income.

The 2010 RCT^24^ intervention compared a modified version of a validated HIV and Hepatitis risk reduction intervention with the standard unmodified intervention as the control. Both intervention and control arms were delivered by peer educators. The standard intervention involved two 60 min sessions 2 weeks apart providing pre-test counselling on HIV, hepatitis B and C, transmission routes, risky drug use, unsafe sex practices, male and female condom use, disinfection of injection equipment and the benefits of drug treatment. Modification of the standard intervention was based on data from a series of focus groups with SSW and the changes to content and language were intended to make the intervention more accessible and specific to SSWs. The SSW specific version included additional coverage of the risks of unprotected oral sexual activity, as well as management of violent victimisation, which were issues highlighted by SSWs in the focus groups.

#### Motivational interviewing based

A single 30 min one-to-one MI-based session about readiness to change^27^ with a trained interviewer completing a change plan worksheet with the participant, using the participant's own words, was intended to increase intrinsic motivation to change. The interviewer used a readiness ruler to rate importance of, readiness for and confidence about suggested changes. The rulers were used to assess where on the scale 0–10 the participants rated themselves with respect to change and what would they need to move to a higher score, that is, be more likely to implement change. Participants were given a copy of the worksheet at the end of the interview. This brief adaptation rather than complex interviews, was used, as it is simple to teach and highly replicable.

#### Other aspects of interventions

All interventions facilitated access to services which were not part of the intervention and apart from one of the substitute prescribing interventions which reported results of baseline infection screening,^28^ uptake was not reported. On-site healthcare or supported access to healthcare was provided in all but one of the studies. The jewellery-making intervention only provided access to support around job-seeking and employment.

#### Theoretical basis for interventions

There is strong evidence for the effectiveness of opiate substitution therapy in reducing mortality and morbidity and improving social functioning amongst problem opiate users in general. This effectiveness has been attributed to a number of factors including prevention of the opiate withdrawal symptoms that may underlie involvement in activity directed at funding illegal drug use.

The jewellery-making educational intervention developed the HIV prevention component around the Social Cognitive Theory^30^ and was based on five elements to improve knowledge, enhance self-efficacy, teach skills, improve communication and negotiation skills and practice skills through role play. The jewellery-making component taught new skills and aimed to increase self-efficacy in accessing job-training programmes and employment.

The HIV and hepatitis risk reduction educational intervention was based on the guiding principle that successful HIV intervention models need to be adapted and tailored to particular social contexts to be effective with unique groups. This underpinned the use of focus groups with the target population in modifying an existing intervention by SSWs for SSWs.

The MI-based intervention was based on Motivational Interviewing theory that is intended to evoke intrinsic motivation for change by resolving ambivalence through a client-centred, goal-orientated counselling approach.^31^

### Research challenges

All the studies identified inherent vulnerabilities of SSWs which contributed to the particular challenges of undertaking research with this population. These challenges were reflected in design and delivery of the studies and interventions.

Involvement in sex work and drug use were considered to make SSWs distrustful of authority, which made study recruitment challenging. Targeted recruiting, either through a service which had established contact with SSWs or by using outreach contact to the red light district, was used to make contact. Two studies used peer recruiters to approach SSWs who were working and a third used a trained recruiter who had previous experience of working with SSWs.

Unstable housing, physical and sexual violence,^27^ and living in a drug using environment^28^ were considered to influence the women's lives and were perceived to influence retention in the study,^28^ follow-up^24^ ^27^ and participants’ ability to maintain any reductions in illicit drug use and lifestyle changes achieved. Researchers were flexible about follow-up locations including jail, rape crisis centre, domestic violence refuge and a pregnant drug users’ service. One study described follow-up contact through friends or relatives,^27^ though researchers also spent time in services frequented by SSWs or the red light area^28^ or in order to facilitate contact. Follow-up was less problematic in studies providing opiate substitution treatment.^25^ ^29^

The authors of the studies acknowledged that the use of self-reported outcome measures in a chaotic drug-using population could be unreliable. In two studies,^28^ ^29^ the authors sought to triangulate self-reports with input from other sources, such as other staff, organisations (such as police) and other patients, in order to improve reliability.

Intervention design and delivery took account of the poor health relating to sex work and drug use, as well as pre-existing conditions such as mental health problems that were compounded by inconsistent or non-existent service use. Poor service use was considered to be related to population lifestyle, though the requirement to pay for healthcare in the USA was also considered a barrier. Interventions emphasised access to health and social services which was provided or facilitated as part of the intervention in all studies except the jewellery making project, which emphasised employment.

### Outcome measures

All included studies relied entirely or in part on subjective and self-reported outcomes.

*Primary outcome*: Prescribing-based studies^25^ ^28^ ^29^ assessed illicit drug use through urinalysis to test for presence or absence of illicit drugs in the urine. Two studies^24^ ^27^ used self-reported measures of levels of drug use but based their assessment interviews on previously validated tools.^32^ ^33^ Outcome assessment follow-up was longer in the substitute prescribing-based studies^25^ ^28^ ^29^ (1–18 years) than in non-prescribing studies(3–6 months).^24^ ^26^ ^27^

*Secondary outcomes*: All measures of levels of sex working were self-reported, though two studies^28^ ^29^ described seeking additional objective input from other staff, outside agencies and even other patients. Homelessness was not reported as an outcome by any of the studies, but as a baseline measure in two.^24^ ^26^

### Study quality and risk of bias

All the included studies were at high risk of bias across all domains and outcomes (table 1).

### Effects of interventions

#### Effects of interventions on illicit drug use

All studies reported a positive intervention effect based on a reduction in the levels of illicit drug use between baseline and post intervention measures (table 2). However, the results of the RCT showed no strong evidence of difference between the intervention and control groups at either 3 months (OR 1.17 (95% CI 0.84 to 1.66)) or 6 months (OR 1.14 (95% CI 0.8 to 1.61)) follow-up. The reporting of this study highlighted positive within-group improvements compared to baseline, which the authors interpreted as showing that both the intervention and the control were having an effect. The MI based study^27^ reported evidence of reduction in the percentage of women reporting daily drug use and the jewellery-making study^26^ an increase in percentage of days reported abstinent in the last 30 (table 2). The studies based on substitute prescribing^25^ ^28^ ^29^ reported reductions in positive urine tests for drug use but did not undertake statistical tests of significance.

**Table 2:** Summary of findings

*Prescribing-based interventions*			
	Litchfield (2010)	Bellis (1993)	Gunne (1986)
Number of participants	34	25	34
Study design	Before/after	Before/after	Before/after
**Intervention type**	**Substitute prescribing**	**Substitute prescribing**	**Substitute prescribing**
Levels of illicit drug use	% urines testing positive for non-prescribed drugs At baseline: 87% (95% CI 75.7% to 98.3%) At 12 months: 72% (95% CI 56.91% to 87.09%)	% urines testing positive for non-prescribed drugs At baseline: 80% (95% CI 64.32% to 95.68%) At 12 months: 51% (95% CI 31.4% to 70.6%)	Women with urine samples consistently testing negative for non-prescribed drugs At baseline: 0% (95% CI 0% to 0%) Up to 220 months: 71% (55.75% to 86.25%)
Involvement in sex working	Women reporting sex working At baseline: 100% (95% CI 100% to 100%) At 12 months: 33% (17.19% to 48.81%)	Not reported	Women not involved in sex work At baseline: 0% (95% CI 0% to 0%) Up to 220 months: 71% (55.75% to 86.25%)
Levels of income from sex work		% income from sex work At baseline: 78%(95% CI 61.76% to 94.24%) At 12 months: 20% (95% CI 4.32% to 35.68%)	
Levels of homelessness	Not reported	Not reported	Not reported
*Non prescribing-based interventions*			
	Surrat (2010)	Sherman (2006)	Yahne (2002)
Number of participants	806	50	27
Study design	RCT	Before/after	Before/after
**Intervention type**	**Educational**	**Educational**	**Psychological**
Levels of illicit drug use	3 months: OR 1.17 (0.84 to 1.66) 6 months: OR 1.14 (0.8 to 1.61)	% women reporting daily drug use: At baseline: 76% (95% CI 64.16% to 87.84%) At 3 months: 55% (95% CI 41.21% to 68.79%)	Reported days abstinent in last 30 days At baseline: 15% (95% CI 1.53% to 28.47%) At 4 months: 51% (95% CI 32.14% to 69.86%)
p Value (where given)		0.003	<0.001
Involvement in sex working	3 months: OR 0.944 (0.67 to 1.32) 6 months: OR 1.14 (0.79 to 1.65)	Median clients/month At baseline: 9 At 3 months 3	% days sex worked in last 30 days At baseline: 59% (95% CI 40.45% to 77.55%) At 4 months: 17% (95% CI 2.83% to 31.17%)
p Value (where given)		0.025	<0.0001
Levels of homelessness	Women reporting homelessness at study entry Usual care: 42.9% Intervention group: 41%	27% reported homelessness in 3 months prior to study	Not reported

#### Effects of interventions on involvement in sex work

All the authors reported a positive intervention effect based on a reduction in the levels of sex work, though the RCT^24^ did not provide strong evidence of an effect at 3 (OR 0.944 (95% CI 0.67 to 1.32)) or 6 month follow-up (OR 1.14 (0.79 to 1.65)). The motivational interview^27^ and jewellery-making^26^ interventions showed some evidence of effectiveness (table 2). Studies based on substitute prescribing^25^ ^28^ ^29^ reported reductions in levels of sex working, though did not provide estimates of uncertainty around these (table 2).

## Discussion

Only a small number of eligible studies were identified and they were not considered suitable for meta-analysis. Although we identified a number of approaches to reducing illicit drug use in SSWs, the evidence of benefit from them was weak. However, the review process and the studies identified provide some useful insights.

### Potential for bias in the review process

Though extensive database, organisational and governmental website searching was undertaken to identify published and unpublished studies, extensive searching for observational studies may identify lower quality evidence at high risk of bias.^21^ As the majority of included studies were observational, bias may also have been introduced through increased publication of observational studies with positive effects.^34^

Perceived risk bias through use of a single reviewer to screen titles and abstracts was addressed by including all studies for full text screening if they could not be definitively excluded on the abstract. Screening of full-text articles was undertaken independently by two individuals to reduce the high potential for bias due to broad inclusion criteria and a large number of observational studies.

### Risks of bias in the evidence identified

A range of quantitative study designs were included despite their high risk of bias. The number of RCTs was expected to be low due to the challenges of undertaking research with this population.^35^

There was high and unclear risk of bias across all domains in the studies identified in this review. It is likely that the nature and direction of bias in the included studies would tend to over-estimate effect size, favouring the interventions of interest, although the lack of sensitivity of some outcome measures would tend to reduce the apparent intervention effect.

The intervention effect described in the RCT is in keeping with improvement over time irrespective of the intervention. Only one study^25^ highlighted the potential role of regression to the mean as a possible source of bias.

### Heterogeneity

Narrow study population inclusion criteria were set to ensure studies focused on female SSWs currently engaged in both street sex work and illicit drug use. This approach reduces heterogeneity in the study population but is likely to reduce the number of eligible studies. However, SSWs actively engaged in drug use and sex work may become trapped in a work-score-use cycle^15^ placing them at very high risk of ill-health and therefore best-placed to gain significant health benefit from an effective intervention. Exclusion criteria were set to avoid inclusion of other groups of sex workers, such as male or transgender workers, who may have very different risk and health need profiles, which could reduce intervention effectiveness.

Owing to the complexity of the issues adversely affecting drug treatment outcomes for SSWs and the interdependent nature of negative influences on health, the review authors were interested in any intervention targeting any part of SSWs lives which had a direct or indirect effect on illicit drug use. Differing intervention types, in addition to the variable quality of the studies included, will introduce heterogeneity in intervention effects.

### Implications for practice and future research

The predominantly observational study design and small numbers of participants reflects the challenges of undertaking research with this population. Though more recent research has demonstrated that experimental methodologies at low risk of bias are possible with SSWs^16^ ^17^ useful insights are provided by the reviewed studies into the management of research challenges through flexible and respectful approaches to working with SSWs, based on developing trust and relationships. Many research challenges are mirrored in clinical service delivery to this group. Partnership working and shared solutions would benefit SSWs; not only through improving the quality of available evidence to underpin service development but also through facilitation of getting good quality research into practice. Future research should also seek to increase use of objectively assessed outcome measures and length of follow-up, as dependency and involvement in sex work may run a relapsing and remitting course. The fact that the studies in clinical services had much longer follow-up suggests that researchers working more closely with clinical and statutory services may find this a useful way forward.

The range of interventions identified in the literature is in keeping with the complexity of the issues influencing problematic drug use and involvement in street sex work. Though all of the interventions in this review were reported to have a positive effect on reducing illicit drug use that was not their primary focus and all of the interventions included elements which addressed the broader life issues of participants. This wrap around opportunistic approach to intervention provision, along with the issues highlighted in the qualitative synthesis of research challenges, indicates the multiple needs of this group and the chaotic effects of substance misuse on their lives. Consistent service attendance is problematic, particularly within the context of homelessness,^8^ the work-score-use cycle^15^ and the disruption to daily lives caused by emergency hospital admissions^13^ and involvement with the criminal justice system.^36^ The requirement for linear progression through drug treatment services is likely to be challenging for this group compared to the more flexible approach that focuses on reducing harms through service engagement and safer practices. Thus a complex intervention addressing a range of needs and social factors should be considered in intervention development. Involving sex workers in the design and development of interventions is likely to ensure appropriateness and accessibility of the intervention and will empower a very marginalised group.

Only two of the six studies explicitly based their intervention on theory, and future studies should seek to identify and utilise appropriate theories in intervention development.^37^ Interventions based on theory have not been consistently shown to be more effective, but this may be due to poor selection and application of theory.^38^

Our inclusion criteria were developed to reflect the significant risk-taking and health needs of women in active addiction and regularly engaged in street sex-working. These were set in light of previous research and the lead author's clinical experience of delivering care to SSWs. Internationally agreed definitions of sex work and drug use are currently lacking,^39^ which limits the generalisability and comparability of research findings. Their development would increase the usefulness of individual studies by enabling meaningful comparison of effectiveness of interventions across countries and setting of sex work. This would be particularly beneficial for this population, where research studies are challenging but the potential for health benefit is substantial.

### Conclusions

The quality of the studies included in this review preclude any firm conclusions about the effectiveness of interventions to reduce illicit drug use in SSWs, but this review provides a base from which to consider the design and evaluation of future interventions. More research using robust methodological study designs is possible and is needed, given the lack of evidence to support current policy.
